# P-1452. A Seasonal Influenza Hemagglutinin mRNA-Based Vaccine Induces Humoral and Cellular Immunity Comparable to an Adjuvanted Inactivated Influenza Virus Vaccine

**DOI:** 10.1093/ofid/ofaf695.1638

**Published:** 2026-01-11

**Authors:** Carole Henry, Daniel Makrinos, Runxia Liu, Maria Cavallaro, Brooke Fenderson, Yanbo Sun, Eleanor Astley, Bethany Girard, Wen-Han Yu, Anthony DiPiazza, Jaap Oostendorp, Robert Paris

**Affiliations:** Moderna, Inc., Cambridge, Massachusetts; Moderna, Inc., Cambridge, Massachusetts; Moderna, Inc., Cambridge, Massachusetts; Moderna, Inc., Cambridge, Massachusetts; Moderna, Inc., Cambridge, Massachusetts; Moderna, Inc., Cambridge, Massachusetts; Moderna, Inc., Cambridge, Massachusetts; Moderna, Inc., Cambridge, Massachusetts; Moderna, Inc., Cambridge, Massachusetts; Moderna, Inc., Cambridge, Massachusetts; Moderna, Inc, Cambridge, MA; Moderna, Inc., Cambridge, Massachusetts

## Abstract

**Background:**

Seasonal influenza continues to cause substantial morbidity and mortality worldwide. Vaccines based on mRNA technology have been licensed for the prevention of SARS-CoV-2 and respiratory syncytial virus (RSV) and have the potential to improve current seasonal influenza vaccine approaches, including applications in combination respiratory vaccines, the inclusion of additional antigens, and the shortening of lead times between strain recommendation and vaccine production.

**Methods:**

In this clinical study (NCT05397223), we compared the immunogenicity of an mRNA-based quadrivalent influenza hemagglutinin vaccine (mRNA-1010) with a licensed adjuvanted inactivated influenza virus vaccine (FLUAD) in healthy adults 18 to 75 years of age. For both vaccine compositions, the 2022/2023 seasonal influenza WHO recommendation for cell- (mRNA-1010) and egg-based (FLUAD) quadrivalent vaccines were followed. We evaluated humoral and cellular immune responses to the 4 influenza strains using hemagglutination inhibition (HAI) assays, flow cytometry–based memory B cell (MBC) profiling, and intracellular cytokine staining for T-cell characterization.

**Results:**

Both mRNA-1010 and FLUAD elicited durable and comparable HAI titers up to 12 months after vaccination, which were associated with robust hemagglutinin-specific MBC responses across all 4 influenza vaccine strains. Notably, mRNA-1010 induced higher frequencies of activated CD27⁺CD21⁻ memory B cells specific to H3 when compared with FLUAD, while inducing similar MBC responses to the other 3 strains. In addition, both mRNA-1010 and FLUAD generated strong and comparable hemagglutinin-specific CD4⁺ T-cell responses. A trend towards higher CD8⁺ T-cell responses was observed in mRNA-1010 recipients compared with FLUAD recipients.

**Conclusion:**

Overall, these findings highlight the capacity of an mRNA-based seasonal influenza vaccine to induce robust humoral and cellular immunity similar to an adjuvanted influenza virus vaccine, supporting the potential of the mRNA platform for seasonal influenza vaccination.

**Disclosures:**

Carole Henry, PhD, Moderna, Inc.: Employee|Moderna, Inc.: Stocks/Bonds (Public Company) Daniel Makrinos, MS, Moderna, Inc.: Employee|Moderna, Inc.: Stocks/Bonds (Public Company) Runxia Liu, PhD, Moderna, Inc.: Employee|Moderna, Inc.: Stocks/Bonds (Public Company) Maria Cavallaro, BS, Moderna, Inc.: Employee|Moderna, Inc.: Stocks/Bonds (Public Company) Brooke Fenderson, PhD, Moderna, Inc.: Employee|Moderna, Inc.: Stocks/Bonds (Public Company) Yanbo Sun, PhD, Moderna, Inc.: Employee|Moderna, Inc.: Stocks/Bonds (Public Company) Eleanor Astley, MSc, Moderna, Inc.: Employee|Moderna, Inc.: Stocks/Bonds (Public Company) Bethany Girard, Ph.D., Moderna, Inc.: Employee|Moderna, Inc.: Stocks/Bonds (Public Company) Wen-Han Yu, PhD, Moderna, Inc.: Employee|Moderna, Inc.: Stocks/Bonds (Public Company) Anthony DiPiazza, PhD, Moderna, Inc.: Employee|Moderna, Inc.: Stocks/Bonds (Public Company) Jaap Oostendorp, PhD, Moderna, Inc.: Employee|Moderna, Inc.: Stocks/Bonds (Public Company) Robert Paris, MD, Moderna, Inc.: Employee|Moderna, Inc.: Stocks/Bonds (Public Company)

